# Thymol and carvacrol induce autolysis, stress, growth inhibition and reduce the biofilm formation by *Streptococcus mutans*

**DOI:** 10.1186/s13568-017-0344-y

**Published:** 2017-02-23

**Authors:** Shams Tabrez Khan, Merajuddin Khan, Javed Ahmad, Rizwan Wahab, Omar H. Abd-Elkader, Javed Musarrat, Hamad Z. Alkhathlan, Abdulaziz A. Al-Kedhairy

**Affiliations:** 1 Zoology Department, College of Science, King Saud University, Riyadh, 11451 Saudi Arabia; 2 DNA Research Chair, Zoology Department, College of Science, King Saud University, Riyadh, 11451 Saudi Arabia; 3 Department of Chemistry, College of Science, King Saud University, Riyadh, 11451 Saudi Arabia; 4 Department of Ag. Microbiology, Faculty of Agricultural Sciences, AMU, Aligarh, 202002 India

**Keywords:** Thymol, Carvacrol, Oral hygiene, Alternative antimicrobials, *S*. *mutans*

## Abstract

Organic compounds from plants are an attractive alternative to conventional antimicrobial agents. Therefore, two compounds namely M-1 and M-2 were purified from *Origanum vulgare* L. and were identified as carvacrol and thymol, respectively. Antimicrobial and antibiofilm activities of these compounds along with chlorhexidine digluconate using various assays was determined against dental caries causing bacteria *Streptococcus mutans*. The IC_50_ values of carvacrol (M-1) and thymol (M-2) against *S*. *mutans* were 65 and 54 µg/ml, respectively. Live and dead staining and the MTT assays reveal that a concentration of 100 µg/ml of these compounds reduced the viability and the metabolic activity of *S*. *mutans* by more than 50%. Biofilm formation on the surface of polystyrene plates was significantly reduced by M-1 and M-2 at 100 µg/ml as observed under scanning electron microscope and by colorimetric assay. These results were in agreement with RT-PCR studies. Wherein exposure to 25 µg/ml of M-1 and M-2 showed a 2.2 and 2.4-fold increase in Autolysin gene (*Atl*E) expression level, respectively. While an increase of 1.3 and 1.4 fold was observed in the super oxide dismutase gene (*sod*A) activity with the same concentrations of M-1 and M-2, respectively. An increase in the *ymc*A gene and a decrease in the *gtf*B gene expression levels was observed following the treatment with M-1 and M-2. These results strongly suggest that carvacrol and thymol isolated from *O. vulgare* L. exhibit good bactericidal and antibiofilm activity against *S*. *mutans* and can be used as a green alternative to control dental caries.

**Electronic supplementary material:**

The online version of this article (doi:10.1186/s13568-017-0344-y) contains supplementary material, which is available to authorized users.

## Introduction

The oral cavity is a complex microbial environment hosting around 600 different bacterial species and many of these bacteria are now being associated with oral diseases (Dewhirst et al. 2010; Moore and Moore 1994; Wade 2013). Dental diseases incur substantial economic losses globally accounting to 298 billion US dollar per year which is 4.6% of the total global health expenditure (Listl et al. 2015). The evidence is also now available that the recurring infections of these oral microorganisms result in a number of systemic diseases further adding to the economic losses and loss of life (Khan et al. 2015; Li et al. 2000). One of the most important etiological agents of dental caries is *Streptococcus mutans* (Loesche 1986). In addition to being classically associated with dental caries, this bacterium also causes other systemic diseases such as ulcerative colitis, endocarditis and septicemia (Kojima et al. 2012; Nobbs 2016; Robbins et al. 1977; Tunkel and Sepkowitz 2002). The biofilm formation by *S*. *mutans* and its ability to promote the biofilm formation by other oral bacteria also makes it more difficult to treat the infections of *S*. *mutans* (Ahn et al. 2008; Klein et al. 2015; Krzyściak et al. 2014). Therefore, for a good oral hygiene, it is very important to control the growth of *S*. *mutans* in the oral cavity. Furthermore, the unwarranted and overuse of the antibiotics in dentistry has resulted in the drug resistance among commensal as well as pathogenic bacteria of the oral cavity including *S*. *mutans* (Leistevuo et al. 2000; Sweeney et al. 2004). Therefore, alternatives to conventional antimicrobial agents are highly and urgently required. Among, the alternative antimicrobials are engineered nanomaterials, plant-based materials, bacteriocins and phage-based therapies (Allen et al. 2014; Khan et al. 2016b, c; Rose et al. 2014).

Plant based materials, especially from edible plants, are an effective alternative antimicrobials especially those classified as GRAS (generally recognized as safe) (Burdock and Carabin 2004). Two such commonly used edible plants in traditional medicine since ancient times are *Origanum* and *Thymus* (Craig 1999; Nostro and Papalia 2012). Essential oils from these plants have been shown to exhibit significant antimicrobial activities and their antimicrobial activity against food-borne pathogens have been already tested (Chorianopoulos et al. 2004). The major components in the essential oils of *Origanum* and *Thymus* are the phenolic monoterpene carvacrol and its isomeric form Thymol (Chorianopoulos et al. 2004; Fachini-Queiroz et al. 2012). Both Carvacrol and Thymol have been classified as GRAS and their use in food has been approved by European Parliament and Council (Hyldgaard et al. 2012). The antimicrobial activity of thymol and carvacrol have been reported against foodborne pathogens such as *Clostridium perfringens*, *Escherichia coli* O157:H7 and *Listeria innocua* (Du et al. 2015; Guarda et al. 2011). The mechanism behind this antibacterial activity includes permeabilization and depolarization of the cytoplasmic membrane, by reducing the pH gradient across the cytoplasmic membrane. This reduction in pH gradient also adversely affects the proton motive force leading to the depletion of the intracellular ATP subsequently leading to cell death (Ultee et al. 2002; Xu et al. 2008). It is also concluded in the same study that the phenolic hydroxyl group of thymol and carvacrol plays an important role in its antimicrobial activity (Ultee et al. 2002).

In this paper, we report a detailed study on antimicrobial and antibiofilm activities of two natural compounds M-1 and M-2 isolated from *O. vulgare* L. Moreover, comparison of antimicrobial and antibiofilm activities of these two compounds with chlorhexidine digluconate a commercially used antiplaque compound and clove oil is also discussed in detail in this study.

## Materials and methods

### Plant material and isolation of essential oils

The aerial parts of *O. vulager* L. grown in two different agro-climatic conditions were collected from Saudi Arabia and Jordan in the month of March 2014 and identified by Dr. Jacob Thomas Pandalayil (a botanical taxonomist from the Herbarium Division, College of Science, King Saud University, Riyadh, KSA). Stems of *O. vulgare* L. aerial parts from Saudi Arabia and Jordan were separated and subjected to hydro-distillation separately in a conventional Clevenger-type apparatus for 3 h according to the methods described previously (2016) to give light yellow colour oils with oil yield of 0.40 and 0.24% respectively.

### Bacterial strains and culture media

The culture of *Streptococcus mutans* ATCC 25175 was purchased from American Type Culture Collection (ATCC). Brain heart infusion broth (BHI; Mast Group, Bootle, UK) with 2% sucrose was used for routine culture. For longer preservation and storage, the strain was stored at −80 °C in 20% glycerol.

### Antimicrobial activity of M-1 and M-2 against *S*. *mutans*

The antimicrobial activity of M-1 and M-2 were determined by two methods namely microdilution method and plate count method. For microdilution method, 90 µl of BHI broth with 2% sucrose was added to each well in a 96 well plate. M-1, M-2, and clove oil were added to final concentrations of 1000, 500, 250, 125, 62.5, 31.25, 15.6 and 0 µg/ml. While, as a positive control chlorhexidine digluconate was added to final concentrations of 10.0, 5.0, 2.5, 0.125, 0.0625, 0.031, 0.015 and 0 µg/ml. To these wells 10 µl of *S*. *mutans* ATCC 25175 cultures grown overnight in BHI broth were added. And the 96 well plate was incubated at 37 °C for 24 h.

For plate count assays M-1, M-2 and clove oil were added to tubes containing 5 ml BHI broth with 2% sucrose to final concentrations of 200, 100, 50 and 25 µg/ml. Aliquots of 500 µl from the cultures of *S*. *mutans* ATCC 25175 grown to log phase were added to these tubes. Chlorhexidine digluconate was added to final concentrations of 10.0, 5.0 and 0 µg/ml as a positive control. Tubes without any test compound were used as a control. The cultures were incubated at 37 °C on a shaker with 150 rpm for 12 h. Following incubation, appropriate dilutions of treated and untreated cells were spread on BHI agar plates. The plates were incubated at 37 °C for 3 d. Finally, colony-forming units (CFUs) were counted and recorded.

### Assessing bacterial viability by MTT assay

The change in bacterial viability after treatment with test compounds was determined using MTT assay as described by Mshana et al. (1998) *S*. *mutans* ATCC 25175 was grown in BHI broth containing 25, 50, 100 and 200 µg/ml of M-1, M-2, and clove oil for 4–6 h at 37 °C. Cells grown with various concentrations of chlorhexidine digluconate (2.5, 5, 10 and 20 µg/ml) were taken as positive control. An aliquot of 40 µl from this culture was transferred to wells in 96 well plates in triplicates. Ten microliters of the MTT (Sigma, USA) solution in PBS (5 mg/ml, pH 7.0) was then added to each well containing bacterial cells. Plates were incubated at 37 °C for 4 h. Following incubation 50 µl of a lysis buffer (20% sodium dodecyl sulfate in 50% N, N-dimethylformamide; pH 4.7) was added to the wells and the plates were further incubated at 37 °C for overnight. Finally, the mixture in the wells was mixed by pipetting and Absorbance at 570 nm was measured using Multiskan microtitre plate reader (Multiskan Ascent, Labsystems, Finland).

### Live and dead staining

*Streptococcus mutans* ATCC 25175 was grown either with 25 µg/ml of M-1, M-2, and clove oil or with 2.5 µg/ml of chlorhexidine digluconate for 8 h at 37 °C on a shaker. Following treatment with test compounds, cells were harvested by centrifugation at 900×*g* and were washed with PBS buffer once, and finally suspended again in sterile PBS buffer (pH 7.5). Live and dead staining was performed using LIVE/DEAD^®^ BacLight™ Bacterial Viability Kit (Molecular Probes, Life technologies, USA) following the instructions of the manufacturer. Cells stained as above were observed under a fluorescence microscope (Nikon Eclipse 80i; Nikon Co., Japan) and cells appearing red and green in colour were recorded as dead and total cells, respectively. Ten fields were counted for each treatment and the percentage of dead cells was calculated by comparing the population of dead cells to total cells in the same field.

### Quantitative assessment of biofilm formation

The standard protocol of Burton et al. (2007) was used to quantitate the inhibition of biofilm formation on 48 well polystyrene plates (Nunc, Denmark) in the presence of the test compounds. In each well 900 µl of sterile BHI medium and 100 µl of exponentially growing cells of *S*. *mutans* ATCC 25175 were added. M-1, M-2, and clove oil were added to final concentrations of 200, 100, 50 and 25 µg/ml. As a positive control chlorhexidine digluconate was added to a final concentration of 10, 5, 2.5 and 1.2 µg/ml. Cultures without any test compounds were taken as control. Plates were then incubated for 24 h at 37 °C. Following incubation medium and the floating cells were gently and completely removed using a micropipette. The biofilm at the bottom of the wells was gently washed with PBS buffer (pH 7.4) three times. Biofilms were stained with 0.4% crystal violet (500 µl) dye for 15 min at room temperature. Wells were washed gently 3 times with PBS buffer to remove unbound dye. The crystal violet retained by the biofilm was solubilized in 500 µl of 33% acetic acid. Finally, absorption was determined at 620 nm using a microtitre plate reader (Multiskan, Finland).

### Scanning electron microscopy of biofilm inhibition

Biofilms on the surface of polystyrene plates were grown in the presence of test compounds as detailed above. For biofilm formation assay *S*. *mutans* was grown with 100 µg/ml of clove oil, M-1, and M-2. Culture medium was removed with the help of a micropipette and the biofilm was washed gently with PBS as described above. The biofilms were stained with the fumes of 1% OsO_4_ at room temperature for 3 h and were rinsed three times with milli-Q. Samples were completely dehydrated with series of ethanol (30, 50, 70, 80, 90, 95 and 100%) and these dehydrated samples were sputter coated with platinum using an SPI-Module line of modular sputter coater. Samples were then observed under Scanning electron microscope (JSM-6380; JEOL), at an accelerating voltage of 10-KV and a magnification of 10,000×. Ten fields for each sample were recorded and compared.

### Quantitative real-time PCR

For quantitative real-time PCR six genes involved in cell death, oxidative stress and biofilm formation were used to assess the change in their expression level following the exposure to the test compounds. Genes targeted in this study include autolysin gene *Atl*A and *Atl*E like autolysin gene, involved in cell wall remodeling and cell autolysis (Ahn and Burne 2006; Ajdić et al. 2002). Other genes targeted in this study include general stress response genes, biofilm formation genes, Polyribonucleotide nucleotidyl transferase (*Pnp*A), superoxide dismutase (SOD), *ymc*A and *gtf*B gene (Carabetta et al. 2013; Yamamoto et al. 2000). Primers used to target these genes are listed in Table 1. The effectiveness of the primers was checked by PCR amplification of the genes using the genomic DNA from *S*. *mutans*. Furthermore, the PCR products obtained from these primers were sequenced using the same primers to confirm the specific amplification of the target genes. Cells were grown in the presence of 15 µg/ml of clove oil, M-1 and M-2 for 12 h at 37 °C. While, *S*. *mutans* cells grown with 1 µg/ml of chlorhexidine digluconate was taken as positive control. Instructions of manufacturer was followed for the extraction of total RNA from treated and untreated cells using RNeasy protect bacteria mini Kit (catalogue no.74524, Qiagen,). The concentration of the extracted RNA was determined using Nanodrop 8000 spectrophotometer (Thermo-Scientific, USA). While, the integrity of the prepared RNA was visualized on 1% agarose gel using BioRads gel documentation system (Universal Hood II, BioRad). The synthesis of first strand cDNA was carried out from 1 µg of total RNA by using Ready-To-GoTM RT-PCR beads (GE Healthcare, UK) and the manufacturer’s protocol. For Real-time quantitative PCR (RT-PCRq) SYBR Green I Master (Roche Diagnostics, Switzerland) and Light Cycler^®^ 480 (Roche Diagnostics, Switzerland) were used. Two microliters of the synthesized template cDNA was added to a final volume of 20 µl reaction mixture. The thermal regime used for Real-time PCR cycle included 10 min at 95 °C followed by 40 cycles involving denaturation at 95 °C for 15 s, annealing at 60 °C for 20 s and elongation at 72 °C for 20 s.

**Table 1:** Primers used for RT-PCR in this study

S. no.	Primer name	Seq (5′–3′)	Gene	Product size (~bp)
1	AtlE_F	agctggtcccaaaggaaatc	*Atl*E	200
	AtlE_R	gcctgtgcccaataatcatc		
2	AtlA_F	ggtttggaggcatcaactgt	*Atl*A	138
	AtlA_R	agttggttggatatacgcgg		
3	Stress_F	taagccttacggtgcctttg	*Pnp*A	200
	Stress_R	attaccaacatcgccatcgt		
4	GtfB_F	gccagccaatgttcatcttt	*gtf*B	160
	GtfB_R	gaggcatttccccaaatgta		
5	YmcA_F	gcttttgcaggaacatgaca	*ymc*A	188
	YmcA_R	tggctcgataatcttggaca		
6	SodA39I_F	trcaycatgayaarcaccat	*sod*A	400
	SodA39J_R	arrtartamgcrtgytcccaracrtc		
7	16S_895F	crcctggggagtrcrg	16S rRNA	200
	16S_1100R	agggttgcgctcgttg		

### Statistical analysis

All the tests were performed in triplicate and the data were expressed as the mean ± standard error of at least three independent experiments. A unpaired *t* test of GraphPad software was used to calculate p values. p values that were considered significant for different tests are provided in the figure captions.

## Results

### Isolation and identification of major compounds M-1 and M-2 from essential oils

**Fig. 1:**
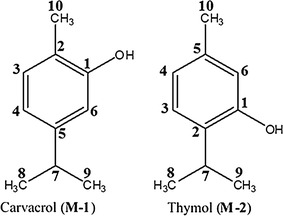
Chemical structure of carvacrol (M-1) and thymol (M-2) (refer to Additional file 1 for raw data)

Essential oils obtained from the stems of *O. vulgare* L. grown in Saudi Arabia and Jordon were analyzed using NMR, GC–MS and GC–FID techniques (results to be published elsewhere) revealed that the composition of the two oils differed significantly. Saudi origanum oil was found to be carvacrol chemotype where carvacrol content was about 80% of the total oil composition. On the other hand, Jordanian origanum oil was identified as thymol chemotype in which the content of thymol was about 69% of the total oil composition. In order to isolate major components, essential oils of Saudi origanum and Jordanian origanum were subjected to preparative thin layer chromatography (pTLC) using chloroform (100%) as developing solvent to give pure compounds M-1 and M-2 from oils of Saudi origanum and Jordanian origanum respectively. The chemical structures of purified compounds M-1 and M-2 were identified as carvacrol and thymol, respectively on the basis of their GC–MS, GC-FID, ^1^H and ^13^C NMR analysis and by comparison of their spectral data with those of related compounds (Fig. 1).

### Antimicrobial activity of test compounds by microdilution method

Antimicrobial activity was determined by using microdilution method. *S*. *mutans* was grown with 1000, 500, 250, 125, 62.5, 31, and 15 and µg/ml of clove oil, M-1, and M-2. Highest antimicrobial activity against *S*. *mutans* ATCC 25175 was observed with thymol (M-2), where almost no growth was observed with 125 µg/ml of M-2 or thymol. While for M-1or carvacrol complete inhibition of growth was observed with 250 µg/ml. Clove oil appeared to be the least effective in inhibiting the growth of *S*. *mutans* wherein even with 1000 µg/ml of concentration complete inhibition of the growth was not observed. Chlorhexidine digluconate which was used as a positive control inhibited the growth of *S*. *mutans* completely at a concentration of 10 µg/ml and was found to be almost twenty times more effective than carvacrol and thymol.

### Antimicrobial activity of test compounds by dilution plating method

**Fig. 2:**
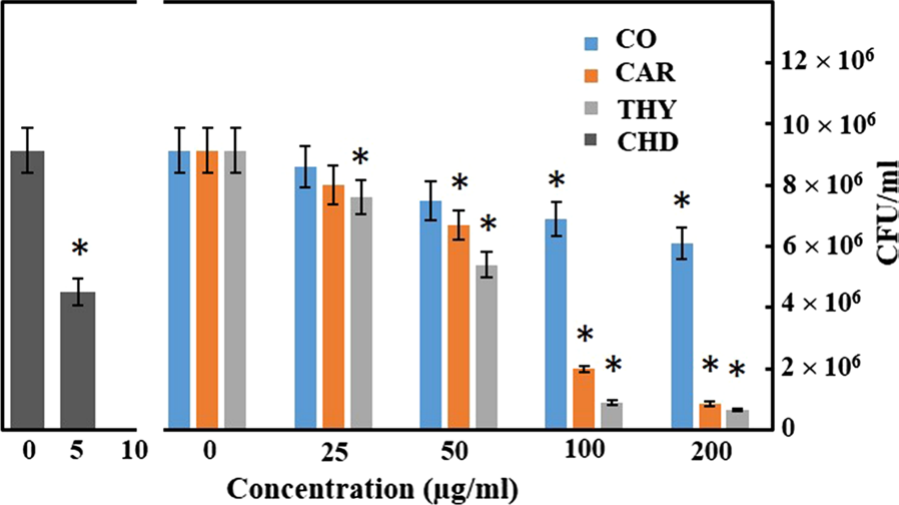
Growth inhibition of *S*. *mutans* in the presence of clove oil (CO), carvacrol (M-1; CAR), thymol (M-2; THY), and chlorhexidine digluconate (CHD). The experiment was done in triplicate and values are presented as mean ± S.D error. *Asterisk* represents the *p* value <0.05 (refer to Additional file 1 for raw data)

Cells of *S*. *mutans* were grown with 200, 100, 50 and 25 µg/ml of clove oil, M-1, and M-2 in BHI broth. Decrease in colony forming units with the increasing concentration of test compounds is shown in Fig. 2. Thymol (M-2) was found to be the most effective against *S*. *mutans* and reduced the population of the bacterium by 93%, at a concentration of 200 µg/ml. While carvacrol (M-1) also reduced the population of *S*. *mutans* by 91% at the same concentration. Clove oil was least effective and could only reduce the population of test bacterium by 33% with the same concentration. The positive control chlorhexidine digluconate reduced the population of *S*. *mutans* by 100% only at a concentration of 10 µg/ml. Clove oil, carvacrol, and thymol exhibited an IC_50_ values of 306, 65 and 54 µg/ml, respectively. While, chlorhexidine digluconate exhibited an IC_50_ value of only 4.9 µg/ml.

### Change in bacterial viability as determined by MTT assay

**Fig. 3:**
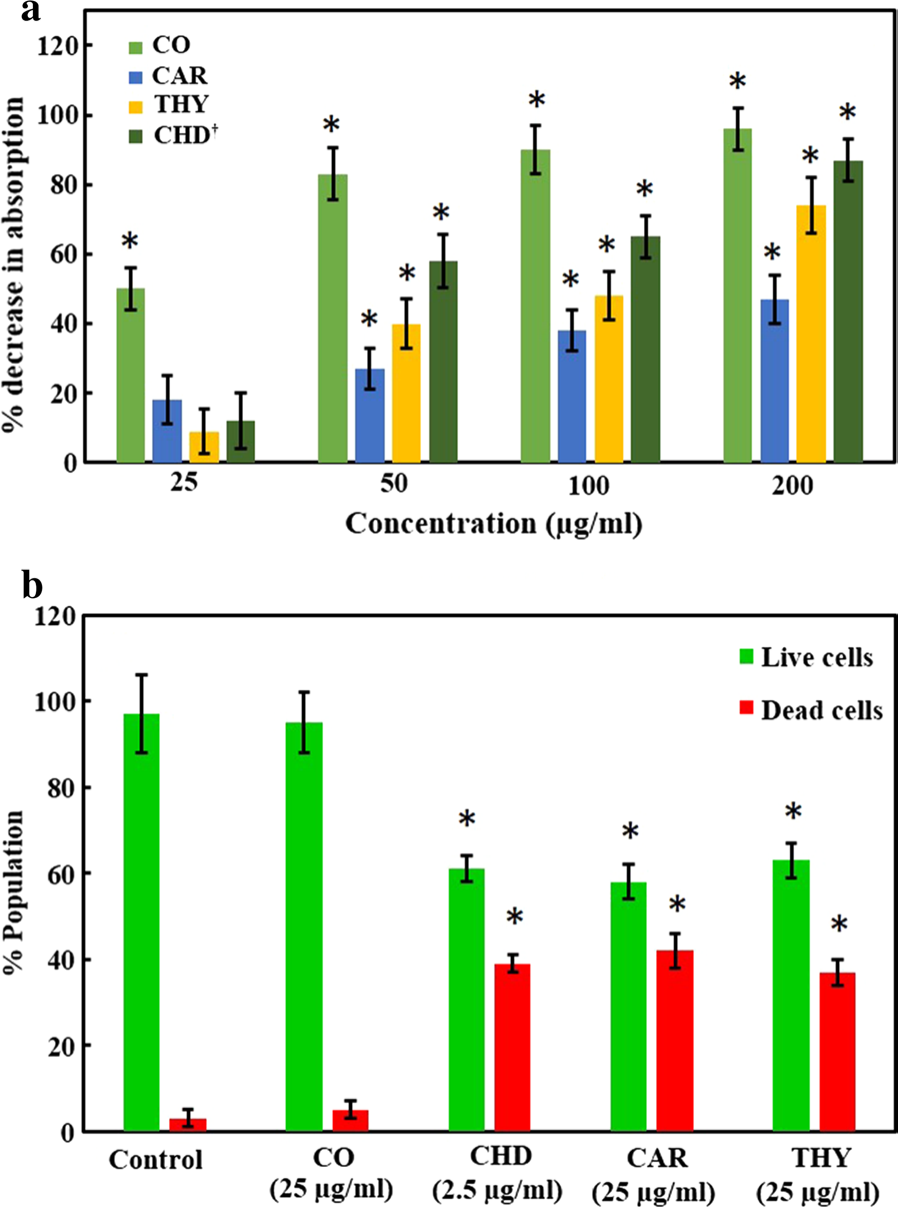
The change in the viability of *S*. *mutans* when grown with clove oil (CO), carvacrol (M-1; CAR), thymol (M-2; THY), and chlorhexidine digluconate (CHD). **a** Shows percent change in the metabolic activity of *S*. *mutans* using MTT assay with various concentrations of test compounds. While **b** shows the percent population of dead and live cells as determined by Live/dead staining when grown with test compounds. The experiment was done in triplicate and values are presented as mean ± S.D error. *Asterisk* represents the *p* value <0.05. *Dagger* the concentration of CHD is 2.5, 5, 10 and 20 µg/ml (refer to Additional file 1 for raw data)

*Streptococcus mutans* was grown with 200, 100, 50 and 25 µg/ml of clove oil, M-1, and M-2 in BHI broth. The formazan formed was dissolved using the lysis solution and the colour was read using a Multiskan spectrophotometer. The change in the viability with different concentrations of the test compounds is shown in Fig. 3a. Thymol (M-2), carvacrol (M-1) and clove oil decreased the cellular activity by 87 ± 6, 74 ± 8 and 47 ± 7%, respectively at a concentration of 200 µg/ml (Fig. 3a). While, chlorhexidine digluconate decreased the cellular activity by 90 ± 7%, at a concentration of 10 µg/ml.

### Change in bacterial viability as determined by dead/live staining

**Fig. 4:**
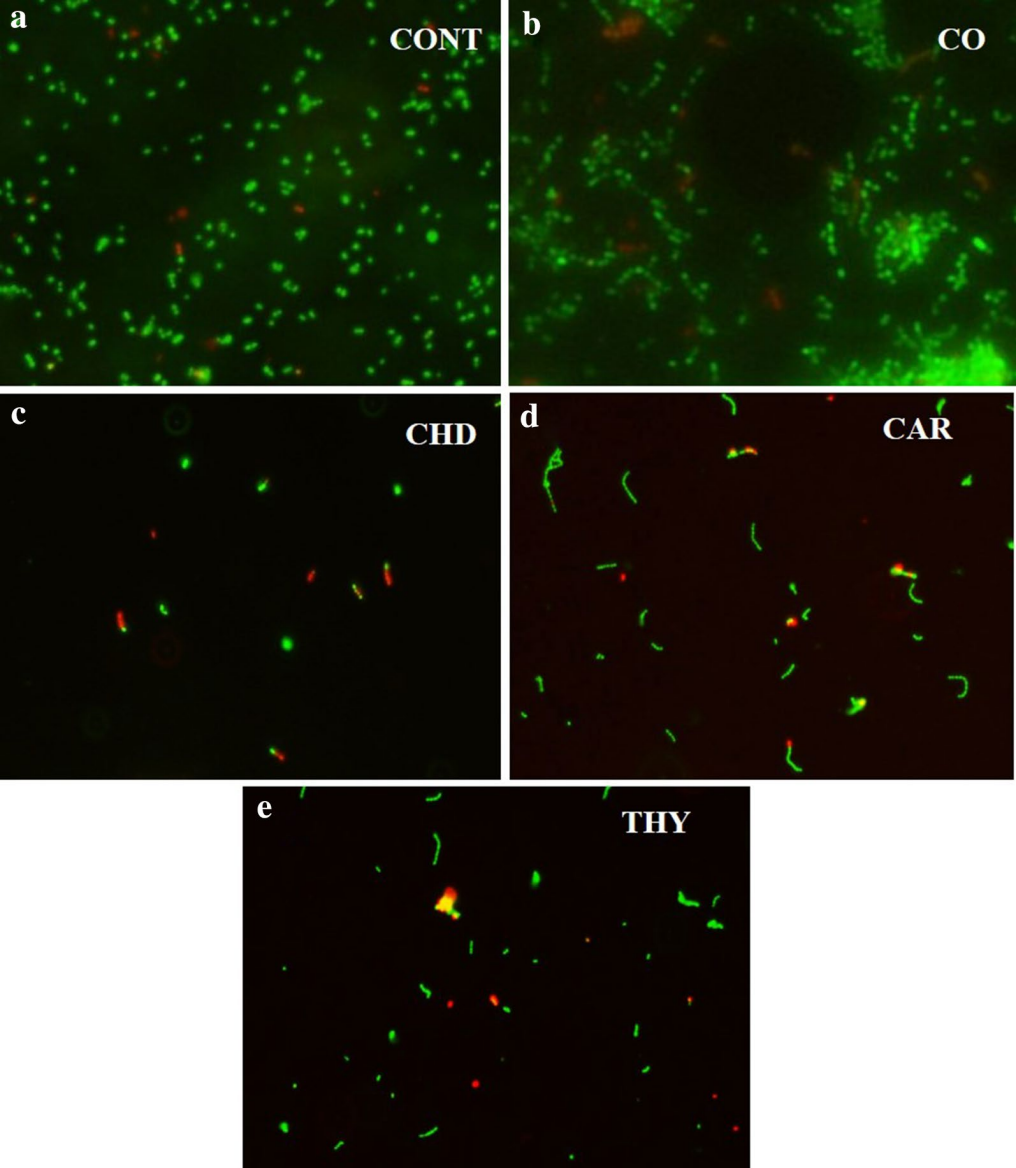
The change in the viability of *S*. *mutans* as determined by Live/dead staining when untreated (**a**, *CONT*), treated with 25 µg/ml of clove oil (**b**, *CO*), carvacrol (**d**, *M-1*) and thymol (**e**, *M-2*) and 2.5 µg/ml of chlorhexidine digluconate (**c**, *CHD*). *Orange* or *red cells* represent dead cells while *green cells* represent live cells (refer to Additional file 1 for raw data)

Live and dead staining was performed after *S*. *mutans* was grown with 25 µg/ml of carvacrol (M-1), thymol (M-2) and clove oil. While cells were also grown with 2.5 µg/ml of chlorhexidine digluconate as a positive control. Thymol (M-2), was found to be the most effective compound which resulted in the death of 42 ± 4% of the cells (Fig. 3b). Carvacrol (M-1) and clove oil resulted in the death of 39 ± 4 and 5 ± 2% of the cells at a concentration of 25 µg/ml. While the positive control chlorhexidine digluconate resulted in the death of 37 ± 3% of the cells at a concentration of 2.5 µg/ml. Figure 4 shows the BacLight staining for live and dead cells after treatment with test compounds. It is clear from the figure that in untreated controls cells appeared more dispersed and were found to be arranged in clusters. Whereas the cells treated with thymol, carvacrol and chlorhexidine appeared in short chains and show significantly low density.

### Quantitative and qualitative assay for biofilm inhibition

**Fig. 5:**
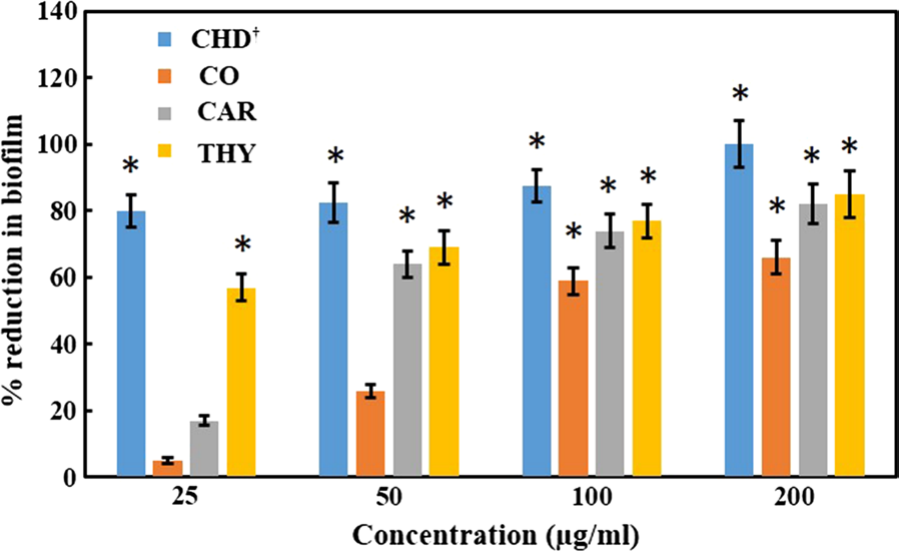
Percent inhibition of the biofilm formation by *S*. *mutans* in the presence of test compounds. *Values* are presented are mean ± S.D error of three independent experiments. *Dagger* chlorhexidine digluconate was tested at a concentration of 2.5, 5, 10 and 20 µg/ml. *Asterisk* represents the *p* value <0.05 (refer to Additional file 1 for raw data)

Crystal violet assay was used for the detection of the biofilm inhibition in the presence of test compounds. Biofilm decreased by 85 ± 7, 82 ± 6 and 66 ± 5% in the presence of 200 µg/ml of thymol (M-2), carvacrol (M-1) and clove oil, respectively (Fig. 5). While the positive control chlorhexidine digluconate reduced the biofilm formation by 87 ± 5% at a very low concentration of 10 µg/ml.

**Fig. 6:**
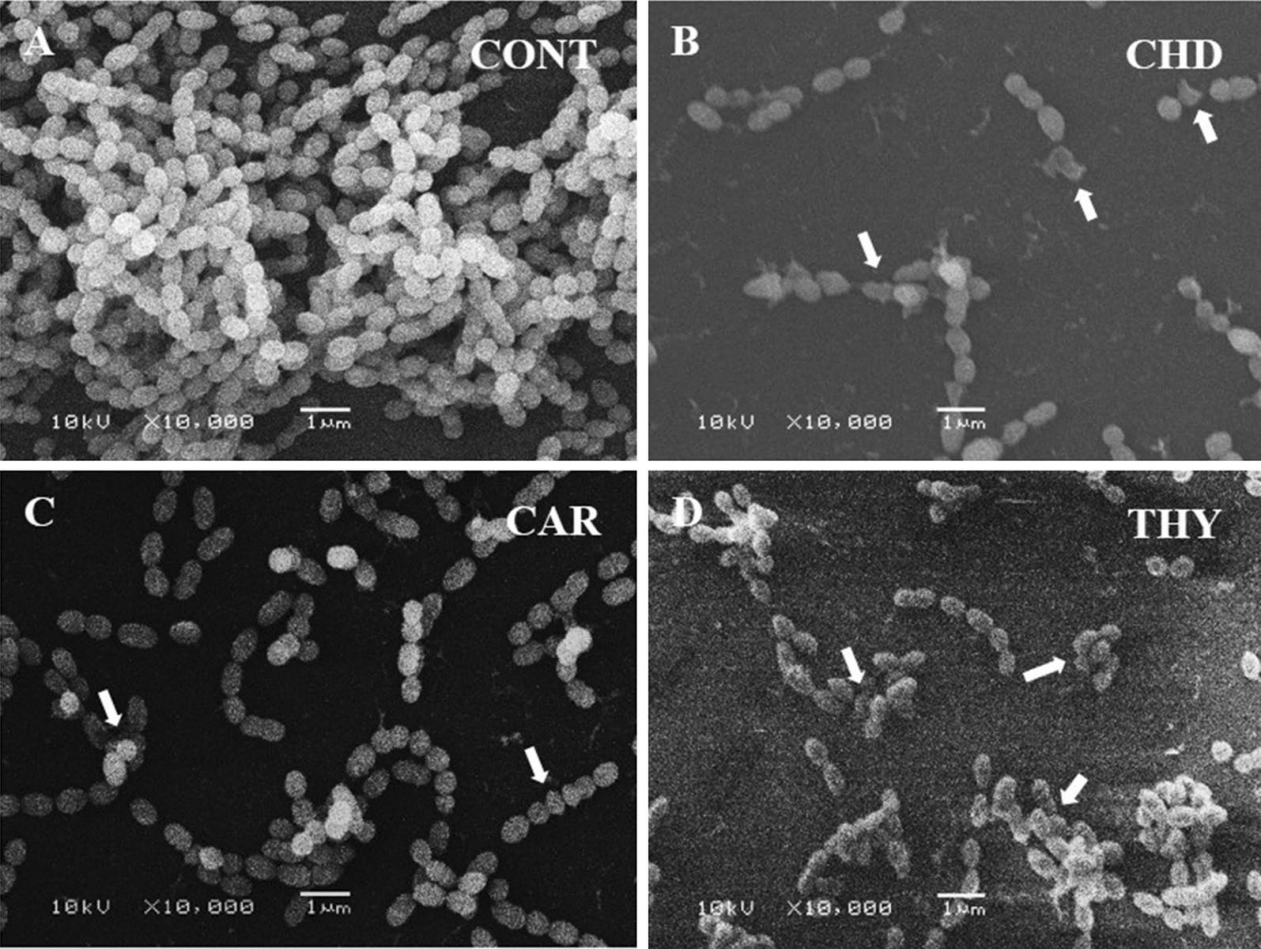
Qualitative assessment of *S*. *mutans* biofilms grown in the presence of test compounds. Control biofilms show high density of cells with homogeneous shape (**A**). While, cells treated with chlorhexidine digluconate (**B**), carvacrol (**C**) and thymol (**D**) show short chains of cells with many deformed and lysed cells marked by *arrows* (refer to Additional file 1 for raw data)

For qualitative assessment, biofilms of *S*. *mutans* grown in 48 well plates in the presence of test compounds or without test compounds were observed under a scanning electron microscope as described in materials and methods. Untreated controls show uniform well grown and densely arranged bunches of cells (Fig. 6A), almost similar pattern was observed for the cells treated with clove oil (data not shown). On the contrary, the cells treated with thymol and carvacrol appeared as short chains with considerably reduced cell density. The cells grown with chlorhexidine show the least density of the cells. Moreover, cells treated with thymol, carvacrol, and chlorhexidine digluconate appeared lysed and deformed and are marked with arrows in Fig. 6 B, C.

### Real-time PCR analysis of the genes involved in cell death, stress and biofilm formation

Change in the expression of six genes namely autolysin like genes *Atl*E (N-acetylmuramoyl-l-alanine amidase type), and *Atl*A like genes, Polyribonucleotide nucleotidyl transferase as a marker of general stress (*Pnp*A), superoxide dismutase gene (SOD), *ymc*A and *gtf*B genes was studied to check the cell death, general stress and biofilm formation activities. Since it was found that two genes *Pnp*A like and *Atl*A like genes were not sufficiently amplified in PCR assay using genomic DNA from *S*. *mutans* as template, these two genes were not used in RT-PCR studies. Furthermore, the sequence of the PCR products amplified using the PCR primers confirms that these primers are specifically binding to the target genes (Additional file 1).

**Fig. 7:**
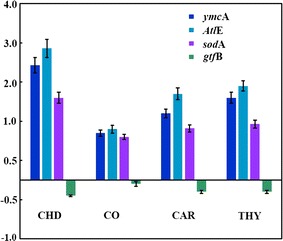
RT-PCR analysis of *S*. *mutans* grown with the test compounds targeting Autolysin *Atl*E, *gtf*B, *sod*A and *ymc*A genes. The expression of *Atl*E, *sod*A and *ymc*A genes was induced by the test compounds while, *gtf*B gene was downregulated (refer to Additional file 1 for raw data)

RT-PCR analysis shows an increase of 2.4, 2.2 and 1.3 folds in the expression of Autolysin gene *Atl*E when *S*. *mutans* was grown with 15 µg/ml of thymol (M-2), carvacrol (M-1) and clove oil, respectively (Fig. 7). This increase in the expression of *Atl*E gene suggests that exposure to thymol and carvacrol induces apoptosis-like activity. Similarly, when *S*. *mutans* was grown with the same concentration of thymol (M-2), carvacrol (M-1) and clove oil, the expression of *ymc*A gene was upregulated by 2.1, 1.7 and 1.2 folds, respectively. The expression level of *sod*A genes also increased by 1.43, 1.32, and 1.1 when grown with 15 µg/ml of thymol (M-2), carvacrol (M-1) and clove oil, respectively. An increase in the expression of these genes shows that exposure to thymol and carvacrol results in an increase in oxidative stress and general stress in *S*. *mutans*. On the contrary the expression level of *gtf*B decreased by 0.3, 0.3 and 0.1 folds, when grown with thymol (M-2), carvacrol (M-1) and clove oil, respectively. This decrease in *gtf*B gene expression suggests the inhibition of biofilm formation which was also observed in other experiments. Similar, trend was observed, with the positive control chlorhexidine digluconate wherein a 2.9, 3.3 and 2.1 folds increase in the expression of *ymc*A, autolysin *Atl*E, and *sod*A genes, respectively was observed at a concentration of 1 µg/ml. While the exposure to chlorhexidine digluconate resulted in 0.4 folds decrease in the expression of the *gtf*B gene.

## Discussion

Essential oils from edible plants especially those classified as GRAS are one of the safe alternatives to traditional antibiotics (Kalemba and Kunicka 2003). But the commercialization of these plant-based products is often hampered by the inability to purify bioactive compounds from these plants. Two such compounds, thymol, and carvacrol are the main constituents of *O. vulgare* L., both compounds are known to exhibit antimicrobial activity against a number of pathogens (Chorianopoulos et al. 2004; Thosar et al. 2013; Ultee et al. 2002). Interestingly, an in vitro study has demonstrated that thymol, and carvacrol show selectively higher antimicrobial activity against the tested pathogenic bacteria (*Escherichia coli*, *Clostridium perfringens*, and *Salmonella*) than the beneficial probiotic bacteria Lactobacillus (Du et al. 2015). When thymol and carvacrol were tested in vivo for their efficacy to inhibit *C*. *perfringens* in broilers it was found that although the population of the pathogen was not reduced significantly but the treatment alleviated intestinal lesions caused by these pathogens (Du et al. 2015). The same group (Du et al. 2016) in their in vivo study on broilers has further demonstrated many beneficial effects of thymol and carvacrol including an increase in feed conversion efficiency, increase in immunity against virus and tumor. It has been reviewed earlier also that herbs that serve as a primary source of these essential oils exhibit anticancer activities (Craig 1999). Hence, thymol and carvacrol in addition to possessing desired antimicrobial activity also have known health benefits making these essential oils a promising alternative antimicrobial agent.

Dental caries and periodontal diseases are most prevalent microbial diseases and *S. mutans* is one of the most important bacterium involved in dental caries (Loesche 1986; Selwitz et al. 2007). To keep a good oral hygiene, it is very important to check the growth of *S*. *mutans*. However, over and unwarranted use of antibiotics has also resulted in the development of antibiotic resistance in these pathogens (Leistevuo et al. 2000). Since, thymol and carvacrol exhibit good antimicrobial activity these essential oils were purified from *O. vulgare* L. and their antimicrobial activity against *S*. *mutans* was determined. Furthermore, the antimicrobial activity of thymol and carvacrol was compared with clove oil, a traditionally used essential oil in dentistry, and with chlorhexidine digluconate a widely used compound in mouthwashes (McBain et al. 2003; Thosar et al. 2013). To our knowledge, this is the first such detailed report on the antimicrobial and antibiofilm activity of thymol and carvacrol against *S*. *mutans*.

IC_50_ values of thymol, carvacrol, clove oil and chlorhexidine digluconate were found to be 54, 65, 306 and 4.9 µg/ml, respectively in this study. These results were further confirmed by MTT assay wherein 200 µg/ml of thymol and carvacrol reduced the cell viability by 87 ± 6 and 74 ± 8%, respectively. While, chlorhexidine digluconate resulted in the death of 37 ± 3% of the cells at a very low concentration of 2.5 µg/ml. In earlier studies, the essential oils from thyme and origanum exhibited high MIC values in a range of 256–512 μg/ml against group A Streptococci (GAS) (Magi et al. 2015). While in the same study commercially available carvacrol exhibited a MIC value in the range of 64–256 µg/ml against GAS (Magi et al. 2015). Decontamination of lettuce using a solution of carvacrol and thymol was also reported since these essential oils are edible (Bagamboula et al. 2004). In another study, the antimicrobial activity of microencapsulated carvacrol and thymol was determined against many microorganisms including foodborne pathogens *Escherichia coli* O157:H7, *Staphylococcus aureus*, and *Listeria innocua*. The MIC of the microencapsulated carvacrol and thymol against these organisms were in the range of 225–375 ppm (Guarda et al. 2011). The positive control chlorhexidine digluconate suppressed the growth and viability of *S*. *mutans* at much lower concentrations as found in earlier studies also (McBain et al. 2003). While, the MIC values of eugenol a main constiuent of clove oil against *S*. *mutans* were reported to be 100 µg/ml (Freires et al. 2015).

As far as the mechanism of the antimicrobial activity is concerned, it is suggested that these compounds are involved in the permeabilization and depolarization of the cytoplasmic membrane. Which results in the reduction of the pH gradient across the cytoplasmic membrane. This lowering of pH gradient leads to the disturbance in proton motive force subsequently leading to the depletion of intracellular ATP level and cell death (Ultee et al. 2002). The increase in the number of dead cells following the treatment with thymol and carvacrol was also observed in this study (Figs. 3b, 4) through propidium iodide staining a dye that can only penetrate into the cells having compromised cell wall. Deformed and lysed cells as observed under scanning electron microscope further confirms that thymol and carvacrol results in the lysis of the cells. The overexpression of autolysin genes involved in the restructuring of the cell wall further confirms these findings. Our results also show the over expression *sod*A and *ymc*A genes, suggesting that treatment with thymol and carvacrol induces general and oxidative stress in the cells. Chlorhexidine digluconate also exhibit its antimicrobial activity primarily through membrane disruption (McBain et al. 2003). Inhibition of glycosidic and proteolytic enzymes by chlorhexidine digluconate is also reported (Hastings 2000). Although carvacrol and thymol also disrupt the cell membrane but these essential oils are not water-soluble which may adversely affect their penetration into the bacterial cells resulting in lower activity. This argument is also supported by the findings that nano form of thymol exhibit better antimicrobial activity than the native form because of the improved dispersibility (Shah et al. 2012).

Another important trait of pathogenicity is biofilm formation. Biofilms exhibit greater resistance to antimicrobial agents and are difficult to treat (Ahn et al. 2008; Curtis et al. 2011). Therefore, the antibiofilm activities of carvacrol (M-1) and thymol (M-2) against *S*. *mutans* was determined. The results of crystal violet assay and the qualitative examination of the biofilms under SEM (Fig. 6) suggest that the two compounds significantly reduce the biofilm formation by *S*. *mutans*. In gene expression studies also the downregulation of glycosyl transferase B (*gtf*B) gene suggests the inhibition of biofilm formation. Previously the antibiofilm activity of thymol and carvacrol against *Pseudomonas aeruginosa* has been reported (Ceylan and Ugur 2015). Chlorhexdine digluconate also reduced the biofilm formation at a concentration of 10 µg/ml. Inhibition of biofilm formation by chlorhexidine has been reported in in vivo studies also (Bailón-Sánchez et al. 2014). As far as the antimicrobial and antibiofilm activities of clove oil are concerned clove oil was found to be least effective.

Results presented in this study strongly suggest that thymol and carvacrol exhibit significant antimicrobial and antibiofilm activities against *S*. *mutans*. Since these compounds are derived from edible plants classified as GRAS, these essential oils can be used in mouthwashes or toothpastes for controlling oral bacteria and for maintaining good oral hygiene. Furthermore, unlike chlorhexidine digluconate, thymol and carvacrol have some health benefits in addition to the desired antimicrobial activity. Although, chlorhexidine digluconate is widely used and do not have any known health hazard but its effect on taste buds is well known (Helms et al. 1995).

## Additional file

**Additional file 1.** Raw data obtained during this study.

## Abbreviations

µg: microgram
µl: microliter
ml: milliliter
nm: nanometer
CAR (M-1): carvacrol
THY (M-2): thymol
CHD: chlorhexidine digluconate
MTT: (3-(4, 5-Dimethylthiazol-2-yl)-2, 5-Diphenyltetrazolium Bromide)
RT-PCR: real time PCR
sodA: super oxide dismutase A
gtf: glucosyltransferase
Atl: N-acetylmuramoyl-l-alanine amidase type
ATCC: American Type Culture Collection
BHI: brain heart infusion broth
°C: degree centigrade
%: percent
CFU: colony-forming unit
h: hour
d: day
s: seconds
rpm: revolutions per minute
PBS: phosphate-buffered saline
OsO4: osmium tetroxide
PCR: polymerase chain reaction
cDNA: complementary DNA
IC50: inhibitory concentration
GC–MS: gas chromatography mass spectrometry
GC–FID: gas chromatography—flame ionization detector
NMR: nuclear magnetic resonance spectroscopy
MIC: minimum inhibitory concentration
ATP: adenosine triphosphate
GRAS: generally recognized as safe

## References

[CR1] Ahn SJ, Burne RA (2006). The *atl*A operon of *Streptococcus mutans*: role in autolysin maturation and cell surface biogenesis. J Bacteriol.

[CR2] Ahn SJ, Wen ZT, Brady LJ, Burne RA (2008). Characteristics of biofilm formation by *Streptococcus mutans* in the presence of saliva. Infect Immun.

[CR3] Ajdić D, McShan WM, McLaughlin RE, Savić G, Chang J, Carson MB, Primeaux C, Tian R, Kenton S, Jia H, Lin S, Qian Y, Li S, Zhu H, Najar F, Lai H, White J, Roe BA, Ferretti JJ (2002). Genome sequence of *Streptococcus mutans* UA159, a cariogenic dental pathogen. Proc Natl Acad Sci USA.

[CR4] Allen HK, Trachsel J, Looft T, Casey TA (2014). Finding alternatives to antibiotics. Ann NY Acad Sci.

[CR5] Bagamboula CF, Uyttendaele M, Debevere J (2004). Inhibitory effect of thyme and basil essential oils, carvacrol, thymol, estragol, linalool and p-cymene towards *Shigella sonnei* and *S*. *flexneri*. Food Microbiol.

[CR6] Bailón-Sánchez ME, Baca P, Ruiz-Linares M, Ferrer-Luque CM (2014). Antibacterial and anti-biofilm activity of AH plus with chlorhexidine and cetrimide. J Endod.

[CR7] Burdock GA, Carabin IG (2004). Generally recognized as safe (GRAS): history and description. Toxicol Lett.

[CR8] Burton E, Yakandawala N, LoVetri K, Madhyastha MS (2007). A microplate spectrofluorometric assay for bacterial biofilms. J Ind Microbiol Biotechnol.

[CR9] Carabetta VJ, Tanner AW, Greco TM, Defrancesco M, Cristea IM, Dubnau D (2013). A complex of YlbF, YmcA and YaaT regulates sporulation, competence and biofilm formation by accelerating the phosphorylation of Spo0A. Mol Microbiol.

[CR10] Ceylan O, Ugur A (2015). Chemical composition and anti-biofilm activity of *Thymus sipyleus* BOISS. subsp. *sipyleus* BOISS. var. davisianus RONNIGER essential oil. Arch Pharm Res.

[CR11] Chorianopoulos N, Kalpoutzakis E, Aligiannis N, Mitaku S, Nychas G-J, Haroutounian SA (2004). Essential oils of Satureja, Origanum, and Thymus species: chemical composition and antibacterial activities against foodborne pathogens. J Agric Food Chem.

[CR12] Craig WJ (1999). Health-promoting properties of common herbs. Am J Clin Nutr.

[CR13] Curtis MA, Zenobia C, Darveau RP (2011). The relationship of the oral microbiotia to periodontal health and disease. Cell Host Microbe.

[CR14] Dewhirst FE, Chen T, Izard J, Paster BJ, Tanner AC, Yu WH, Lakshmanan A, Wade WG (2010). The human oral microbiome. J Bacteriol.

[CR15] Du E, Gan L, Li Z, Wang W, Liu D, Guo Y (2015). In vitro antibacterial activity of thymol and carvacrol and their effects on broiler chickens challenged with *Clostridium perfringens*. J Anim Sci Biotechnol.

[CR16] Du E, Wang W, Gan L, Li Z, Guo S, Guo Y (2016). Effects of thymol and carvacrol supplementation on intestinal integrity and immune responses of broiler chickens challenged with *Clostridium perfringens*. J Anim Sci Biotechnolo.

[CR17] Fachini-Queiroz FC, Kummer R, Estevão-Silva CF, Carvalho MD, Cunha JM, Grespan R, Bersani-Amado CA, Cuman RK (2012). Effects of thymol and carvacrol, constituents of *Thymus vulgaris* L. essential oil, on the inflammatory response. Evid Based Complement Alternat Med.

[CR18] Freires IA, Denny C, Benso B, de Alencar SM, Rosalen PL (2015). Antibacterial activity of essential oils and their isolated constituents against cariogenic bacteria: a systematic review. Molecules.

[CR19] Guarda A, Rubilar JF, Miltz J, Galotto MJ (2011). The antimicrobial activity of microencapsulated thymol and carvacrol. Int J Food Microbiol.

[CR20] Guarda A, Rubilar JF, Miltz J, Galotto MJ (2011). The antimicrobial activity of microencapsulated thymol and carvacrol. Int J Food Microbiol.

[CR21] Hastings DC, Newman HN, Wilson M (2000). Non-antibiotic plaque chemotherapy. Dental plaque revisited: oral biofilms in health and disease.

[CR22] Helms JA, Della-Fera MA, Mott AE, Frank ME (1995). Effects of chlorhexidine on human taste perception. Arch Oral Biol.

[CR23] Hyldgaard M, Mygind T, Meyer RL (2012). Essential oils in food preservation: mode of action, synergies, and interactions with food matrix components. Front Microbiol.

[CR24] Kalemba D, Kunicka A (2003). Antibacterial and antifungal properties of essential oils. Curr Med Chem.

[CR25] Khan ST, Al-Khedhairy AA, Musarrat J (2015). ZnO and TiO_2_ nanoparticles as novel antimicrobial agents for oral hygiene: a review. J Nanopart Res.

[CR26] Khan M, Al-Saleem MSM, Alkhathlan HZ (2016). A detailed study on chemical characterization of essential oil components of two *Plectranthus* species grown in Saudi Arabia. J Saudi Chem Soci.

[CR27] Khan ST, Ahmad J, Ahamed M, Musarrat J, Al-Khedhairy AA (2016). Zinc oxide and titanium dioxide nanoparticles induce oxidative stress, inhibit growth, and attenuate biofilm formation activity of *Streptococcus mitis*. J Biol Inorg Chem.

[CR28] Khan ST, Musarrat J, Al-Khedhairy AA (2016). Countering drug resistance, infectious diseases, and sepsis using metal and metal oxides nanoparticles: current status. Coll Surf B Biointerfaces.

[CR29] Klein MI, Hwang G, Santos PHS, Campanella OH, Koo H (2015). *Streptococcus mutans*-derived extracellular matrix in cariogenic oral biofilms. Front Cell Infect Microbiol.

[CR30] Kojima A, Nakano K, Wada K, Takahashi H, Katayama K, Yoneda M, Higurashi T, Nomura R, Hokamura K, Muranaka Y, Matsuhashi N, Umemura K, Kamisaki Y, Nakajima A, Ooshima T (2012). Infection of specific strains of *Streptococcus mutans*, oral bacteria, confers a risk of ulcerative colitis. Sci Rep.

[CR31] Krzyściak W, Jurczak A, Kościelniak D, Bystrowska B, Skalniak A (2014). The virulence of *Streptococcus mutans* and the ability to form biofilms. Eur J Clin Microbiol Infect Dis.

[CR32] Leistevuo J, Järvinen H, Österblad M, Leistevuo T, Huovinen P, Tenovuo J (2000). Resistance to mercury and antimicrobial agents in *Streptococcus mutans* isolates from human subjects in relation to exposure to dental amalgam fillings. Antimicrob Agents Chemother.

[CR33] Li X, Kolltveit KM, Tronstad L, Olsen I (2000). Systemic diseases caused by oral infection. Clin Microbiol Rev.

[CR34] Listl S, Galloway J, Mossey PA, Marcenes W (2015). Global economic impact of dental diseases. J Dent Res.

[CR35] Loesche WJ (1986). Role of *Streptococcus mutans* in human dental decay. Microbiol Rev.

[CR36] Magi G, Marini E, Facinelli B (2015). Antimicrobial activity of essential oils and carvacrol, and synergy of carvacrol and erythromycin, against clinical, erythromycin-resistant Group A Streptococci. Front Microbiol.

[CR37] McBain AJ, Bartolo RG, Catrenich CE, Charbonneau D, Ledder RG, Gilbert P (2003). Effects of a chlorhexidine gluconate-containing mouthwash on the vitality and antimicrobial susceptibility of in vitro oral bacterial ecosystems. Appl Environ Microbiol.

[CR38] Moore WEC, Moore LVH (1994). The bacteria of periodontal diseases. Periodontology.

[CR39] Mshana RN, Tadesse G, Abate G, Miorner H (1998). Use of 3-(4,5-dimethylthiazol-2-yl)-2,5-diphenyl tetrazolium bromide for rapid detection of rifampin-resistant *Mycobacterium tuberculosis*. J Clin Microbiol.

[CR40] Nobbs A (2016). Getting to the heart of the matter: role of *Streptococcus mutans* adhesin Cnm in systemic disease. Virulence.

[CR41] Nostro A, Papalia T (2012). Antimicrobial activity of carvacrol: current progress and future prospectives. Recent Pat Antiinfect Drug Discov.

[CR42] Robbins N, Szilagyi G, Tanowitz HB, Luftschein S, Baum SG (1977). Infective endocarditis caused by *Streptococcus mutans*. A complication of idiopathic hypertrophic subaortic stenosis. Arch Intern Med.

[CR43] Rose T, Verbeken G, Vos DD, Merabishvili M, Vaneechoutte M, Lavigne R, Jennes S, Zizi M, Pirnay JP (2014). Experimental phage therapy of burn wound infection: difficult first steps. Int J Burns Trauma.

[CR44] Selwitz RH, Ismail AI, Pitts NB (2007). Dental caries. Lancet.

[CR45] Shah B, Davidson PM, Zhong Q (2012). Nanocapsular dispersion of thymol for enhanced dispersibility and increased antimicrobial effectiveness against *Escherichia coli* O157:H7 and *Listeria monocytogenes* in model food systems. Appl Environ Microbiol.

[CR46] Sweeney LC, Dave J, Chambers PA, Heritage J (2004). Antibiotic resistance in general dental practice–a cause for concern?. J Antimicrob Chemother.

[CR47] Thosar N, Basak S, Bahadure RN, Rajurkar M (2013). Antimicrobial efficacy of five essential oils against oral pathogens: an in vitro study. Eur J Dent.

[CR48] Tunkel AR, Sepkowitz KA (2002). Infections caused by viridans streptococci in patients with neutropenia. Clin Infect Dis.

[CR49] Ultee A, Bennik MH, Moezelaar R (2002). The phenolic hydroxyl group of carvacrol is essential for action against the food-borne pathogen *Bacillus cereus*. Appl Environ Microbiol.

[CR50] Wade WG (2013). The oral microbiome in health and disease. Pharmacol Res.

[CR51] Xu J, Zhou F, Ji BP, Pei RS, Xu N (2008). The antibacterial mechanism of carvacrol and thymol against *Escherichia coli*. Lett Appl Microbiol.

[CR52] Yamamoto Y, Higuchi M, Poole LB, Kamio Y (2000). Role of the dpr product in oxygen tolerance in *Streptococcus mutans*. J Bacteriol.

